# Providing pharmaceutical care remotely through medicines delivery services in community pharmacy

**DOI:** 10.1016/j.rcsop.2022.100187

**Published:** 2022-10-08

**Authors:** Oisín N. Kavanagh, Aaron Courtenay, Fatimah Khan, Deborah Lowry

**Affiliations:** a School of Pharmacy*,* Newcastle University*,* United Kingdom*.*; b School of Pharmacy and Pharmaceutical Sciences*,* Ulster University*,* Ireland*.*

**Keywords:** Community pharmacy, Delivery services, Home delivery, Pharmacy services, Pharmaceutical care

## Abstract

**Background:**

The delivery of pharmaceutical care – and what that means – has been at the centre of many transformations of the pharmacy profession in the last century. Today, the exponential growth of pharmacies which provide pharmaceutical care exclusively online has placed increased scrutiny on the quality of the care they provide.

**Aim:**

As more patients are managed by remote pharmaceutical care (via medicines delivery services), we sought to critically evaluate this service to identify new research directions.

**Methods:**

The COnsolidated criteria for REporting Qualitative research and Standards for reporting qualitative research guideline provided the methodological framework throughout this process.

**Results:**

We reveal that although home delivery services ensure that many patients have access to their medicines, it may reduce time available to provide comprehensive pharmaceutical care, particularly in traditional brick-and-mortar pharmacies.

**Conclusion:**

We highlight a critical need for research in this area and suggest a variety of research directions: is remote pharmaceutical care a matter of convenience? Does remote pharmaceutical care help patients adhere to their medicines? How do digital health innovations impact care across patient demographics? What does comprehensive pharmaceutical care mean for patients?

## 1 Introduction

Over the last century, the link between reimbursement and dispensing volume has placed significant emphasis on dispensing activities in the practice of pharmacy;^1^ this is at odds with recent developments in pharmaceutical care.^1^ Their utility has led to the revaluation of the traditional reimbursement model, seeking to shift the focus of the pharmacy profession further away from dispensing volume.^1^ These additional services are often not commissioned and there is a critical need to gather evidence to help policy makers. As such, current and emerging services need to be critically evaluated to understand where they fit within these new models of practice.^1^ Home delivery services, is one such service is which has seen considerable expansion in recent years. In general, pharmacies are not funded for this service and often pay for it out of pocket. Accordingly, it is becoming increasingly economically unfeasible as online pharmacies have entered the market. Although pharmacy unions have responded with advertising campaigns encouraging patients to ‘Support Your Local Pharmacy’, multinational corporations loom on the horizon.

The tension between commercial realities and comprehensive patient care in community pharmacy has been highlighted previously, particularly as a barrier to pharmacy practice research.^1^ As such, there exists a research gap in this area. Home deliveries saw significant uptake during the coronavirus pandemic of 2019 (COVID-19) and is expected to continue.^2^ Some authors have argued that they should be evaluated post-pandemic to ensure continuity of care across the spectrum of hospital and community pharmacies.^3^^,^^4^ Modern approaches to pharmacy delivery services have been described^5^ such as disease stratification of home delivery, to design a cost-effective service for specific patient groups.^6^ Association between delivery services and compliance has been identified and there is scope to explore this further through clinical trials.^7^^,^^8^ Critically, the aspect of convenience has also been commented on and one study has highlighted that to improve adherence, health systems are required to address convenience across the care spectrum, which includes home delivery services.^9^ Analysis of pharmacy location and patient demographics has revealed a lack of access for patients who are elderly^10^ and for those who live in areas of socio-economic deprivation.^11^ Other work explores how to reduce errors with pharmacy deliveries using novel methodologies such as six-sigma.^12^

Pharmacy home deliveries for medications and medical supplies exists in various forms within most United Kingdom (UK) pharmacies. In 2015, 56% of pharmacies in England reported that up to 10% of their total dispensing was deliveries and, on average, each delivery consisted of 2.02 items.^13^ In Northern Ireland (NI), the average cost per item dispensed is £9.99, but may be as high as £11.16 in some regions.^13^^,^^14^ These rates may vary based on patient population, geographic location and other factors. As medication delivery can be provided by volunteers, staff with various levels of training, external couriers or by the pharmacist and their team personally, dispensary staff work alongside the pharmacist and delivery driver to ensure that delivery of medications are safe, accurate and adhere to legislative requirements. Much has been written by regulatory organisations on the great professional risk and the complexity surrounding the implementation and maintenance of delivery services, particularly during COVID-19.15., 16., 17., 18, 19. This is particularly important for medical gases, controlled drugs, and drugs that must be temperature controlled. With each pharmacy responsible for developing its own set of guidelines and Standard Operating Procedures (SOPs), this may result in considerable heterogeneity across organisations and may reflect the need for educational standardisation. It is imperative that optimised patient care is achieved by fully understanding what is needed to deliver a high-quality, safe service.

Northern Ireland (NI) has one of the highest prescription rates in western Europe; antibiotic and antidepressant prescribing have been particularly highlighted, with clear indications of high rates of prescribing in areas with significant deprivation.20., 21, 22. This provides an interesting test-bed to explore the limits of remote pharmaceutical care. In this context, we seek to understand what it means to provide home delivery services, to evaluate what kind of care is currently provided and, what are the views of pharmacy teams on the value of this service for their patients.

## 2 Methodology

### 2.1 Setting and design

The study aimed to elicit pharmacy staff experiences, opinions and perceptions of pharmacy home delivery services within Northern Ireland. A qualitative questionnaire was developed to gather information in a convenient and easily distributed format.^23^ This approach was chosen to gather rich, descriptive data on delivery services to compliment the knowledge gained through quantitative methods from prescription databases.^24^

The questionnaire was administered online via Jisc software and was distributed by email to all 553 community pharmacies listed on the Pharmaceutical Society of Northern Ireland's (PSNI) Register, by the PSNI on our behalf. Eligible respondents included all pharmacists (including pre-registration trainees), pharmacy technicians, pharmacy assistants and pharmacy delivery drivers and all groups where encouraged to respond. The pharmacist was responsible for distributing these surveys to their team as some of these groups are not registered. Although we recognise that this could mean that multiple respondents per pharmacy where captured, we do not intend to make generalisations from our data as our goal is to identify areas for further study (this area of practice is rarely explored in the literature). Separate sets of questions were developed for dispensary staff and delivery staff due to the variation in job roles. For dispensary staff, the questionnaire featured 11 multiple-choice, 3 ranked (five-point Likert scale) and 12 open-text questions. For delivery staff, there were 8 multiple-choice, 1 ranked (five-point Likert scale) and 6 open-text questions. All questionnaire responses were anonymous. Part one of the questionnaire contained multiple choice questions that sought to gather respondent demographics and workplace data (i.e., job title, type of workplace, location, distance from GP etc.). Information regarding current delivery services were explored in part two of the questionnaire. The final part of the questionnaire investigated pharmacy staff perspectives regarding home delivery services. Consent was obtained in the form of questionnaire completion and participants were informed of this at the start of the questionnaire.

### 2.2 Data collection and analysis

A total of 38 questionnaires were completed with a mixed group of respondent type. The total sample size is unknown since the questionnaire was distributed to pharmacy owner and manager emails with one prompt after two weeks to encourage dispensary and delivery staff to participate. As of 2020/21 there were 2824 community pharmacists in Northern Ireland, but the number of pharmacy technicians remains unknown as there is no pharmacy technician register. It is also possible that some respondents may work in the same pharmacy, so we are unable to stratify respondents by considering response count against total pharmacies emailed. We framed our analysis around the following themes: pharmaceutical care, balancing commercial and professional demands and, risks and risk management. The questionnaire was emailed in two intervals: one initial email callout and a secondary reminder email callout two weeks later, analysis of the data obtained in the first and second ‘round’ suggested that we reached saturation prior to closing the survey. We infered saturation as no new themes emerged in the second round of responses. Two members of the research team (FK and OK) analysed the data independently via content analysis and the team discussed their findings to generate a set of codes, which were grouped into themes. These themes were revised and reconstructed iteratively until the final set of themes emerged. The COnsolidated criteria for REporting Qualitative research and Standards for reporting qualitative research guideline provided the methodological framework throughout this process.

### 2.3 Ethics statement

Due to the anonymised nature of the reflections herein, this work carries little to no risk to participants. Ethical approval for this study was granted by the Biomedical Sciences Ethics Filter Committee at Ulster University committee who considered all aspects of the project including recruitment, participation, informed consent, and data protection.

## 3 Quantitative results

### 3.1 Survey responses and contextualisation

**Fig. 1:**
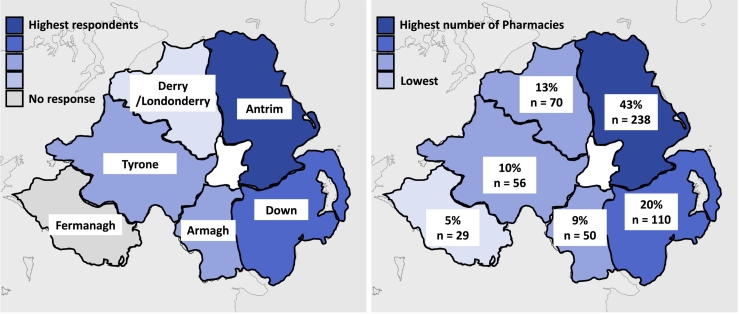
Heat maps illustrating the survey response rate (left) and the total number of pharmacies stratified by county (right).

A total of 38 respondents completed the questionnaire. This included 12 (31.6%) pharmacist employers, 22 (57.9%) pharmacist employees, 1 (2.6%) pre-registration pharmacist, 1 (2.6%) technician and 2 (5.3%) delivery drivers. Of these respondents, 3 (7.9%) work in large multiple pharmacies (100 or more pharmacies), 18 (47.4%) work in small multiples (6–99 pharmacies) and 14 (44.7%) work within independent pharmacies (1–5 pharmacies). The highest proportion of respondent workplaces were located within Country Antrim (44.7%) followed by counties Down (18.4%), Armagh (13.1%), Derry/Londonderry (10.5%) and Tyrone (13.15%). There were no respondents from County Fermanagh. Of these workplaces, 92.1% were located within 5 miles of a GP practice (Fig. 1).

Ninety-three percent of respondent workplaces offered formal delivery services, carried out by dedicated delivery drivers or by a staff member when necessary. Only two (6%) respondent workplaces did not offer a formal delivery service and delivered medications were for a variety of clinical conditions. Most respondents (91.7%) observed notable changes in services due to the COVID-19 pandemic.

**Fig. 2:**
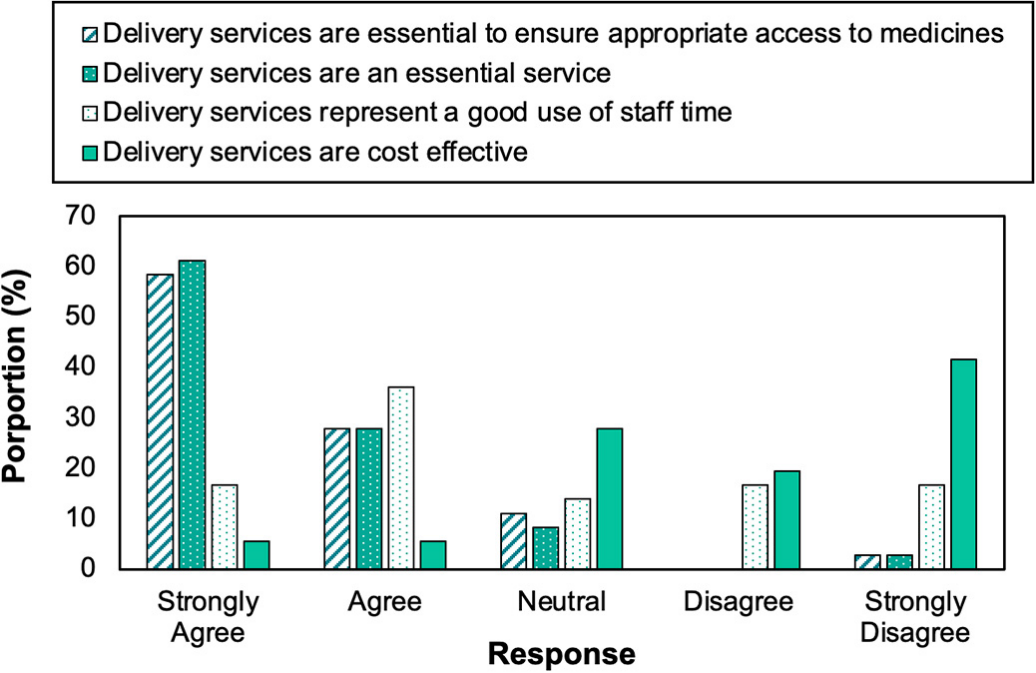
Five-point Likert scale to quantify pharmacy team's perception of the service.

Pharmacy staff were prompted to rank their opinions of delivery services on a five-point Likert scale (Fig. 2). Delivery services were deemed as essential to ensure appropriate access to medicines by 86.1% of respondents, strongly agree (58.3%) and agree (27.8%). Delivery services were also viewed as being an essential service among 88.9% of respondents, strongly agree (61.1%) and agree (27.8%). There were mixed perspectives across the Likert scale on delivery services being a good use of staff time, with only 52.8% agreeing or strongly agreeing, 13.9% neutral and 33.3% disagreeing or strongly disagreeing. Cost efficacy of delivery services were viewed negatively by 61.1% of respondents (disagree or strongly disagree), neutral by 27.8% and positively by 11.1% (agree or strongly agree).

**Fig. 3:**
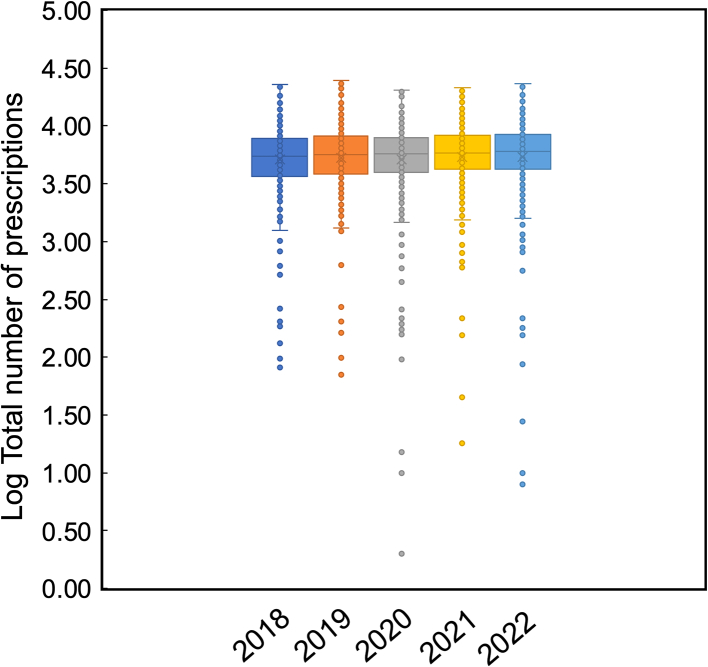
A box plot illustrating NI prescription data for the month of April between 2018 and 2022.

In Northern Ireland, there are 526 pharmacies that dispensed 42.2 million items during 2021–22, predominantly by brick-and-mortar pharmacies.^25^ This equates to an average of 81,482 items per pharmacy.^25^ The top 5 pharmacies all provide online delivery services but also have a base in their respective communities (Fig. 3).^25^ Fermanagh and Omagh have the most pharmacies with 40 pharmacies per 100,000 people in contrast to Lisburn and Castlereagh who have 18 (Table 1).^25^

**Table 1:** Pharmacy distribution across counties in study.^25^

Local Government District	Number of pharmacies	Population	Pharmacies per 100,000 people
Antrim & Newtownabbey	33	145,661	22.8
Ards & North Down	39	163,659	24
Armagh city, Banbridge & Craigavon	48	218,656	21.8
Belfast	128	345,418	37.2
Causeway Coast & Glens	40	141,746	27.6
Derry City & Strabane	44	150,756	29.1
Fermanagh & Omagh	46	116,812	38.9
Lisburn & Castlereagh	27	149,106	18.1
Mid & East Antrim	31	138,994	22.1
Mid Ulster	38	150,293	25.1
Newry, Mourne & Down	52	182,074	28.3

**Fig. 4:**
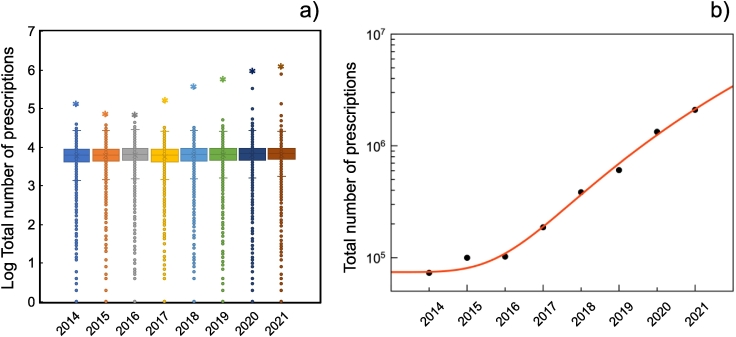
An analysis of NHS England Business Services Authority (NHSBSA) prescription data for the month of April between 2014 and 2021. (a) A box plot to show the quantity of prescriptions dispensed per contractor the top contractor is highlighted with a star and (b) total number of prescriptions dispensed by the top three online pharmacies for this period fitted with least squares to a power law function.

To contextualise current trends in medicines delivery across the UK, an analysis of NHS Business Services Authority (NHSBSA) prescription data is presented in Table 2 and Fig. 4 revealed that the pharmacy with the highest dispensing volume in all years except 2014 (where it was PharmacyPlus, which have since closed) is Pharmacy2U. This contractor exclusively delivers dispensed medicines and was a significant outlier across all the years. Further analysis of the top three online pharmacies (per item dispensed) revealed that online pharmacies are growing exponentially. Indeed, all outliers in the years 2019 onwards are online pharmacies. Additional (commissioned) services offered by the top three online pharmacies are documented in Table 2, this illustrates that although the dispensing activity of these pharmacies have been growing exponentially, additional services do not scale to the same proportion. Figure 4 and Table 2.

**Table 2:** Number of New Medicine Service interventions declared for top three online pharmacies in April between 2014 and 2021 against the total number of prescriptions dispensed.

Year	Number of New Medicine Service interventions declared				Total number of prescriptions (professional fees)			
	*Total*	*Mean*	*Median*	*SD*	*Total*	*Mean*	*Median*	*SD*
2014	0	6	2	10	72,925	6963	6174	4102
2015	0	6	2	9	99,505	7121	6297	4047
2016	0	6	2	10	102,020	7475	6645	4172
2017	0	7	3	10	186,709	6891	6123	4048
2018	6	6	3	10	384,093	7247	6474	5055
2019	0	7	4	11	606,866	7340	6541	6375
2020	0	5	0	9	1,334,956	7667	6775	9888
2021	84	10	6	13	2,100,488	7753	6832	13,964

Standard Deviation (SD), rounded to the nearest whole number.

### 3.2 Qualitative results

#### 3.2.1 Convenient access to healthcare

Many respondents highlighted that delivery services removed barriers, particularly for immobile, elderly patients and those in rural communities.

“*Deliveries add to the patient care we deliver to patient, especially to those who cannot come to the pharmacy e.g., the elderly and house bound*.” (Participant 25)

“*Essential for some elderly patients who may have no other way to get to the pharmacy as we are rural - lack of public transport*.” (Participant 9)

Some respondents noted that in their pharmacy these patients represented a small population of overall deliveries made.

“*Good for vulnerable patients but abused by others who receive multiple deliveries per week with no specific requirement for the service*.” (Participant 29)

“*This is not a professional service and takes up valuable time. People who get delivery don't need it*.” (Participant 28)

Further responses suggested that this service is offered mainly to increase commercial competitiveness through convenience with the added benefit of assisting venerable patients who also avail of the service too.

“*More patients are willing to get their prescription dispensed with us because of convenience*.” (Participant 34)

During COVID an advertising campaign promoted the use of delivery services and provided funding for community pharmacies to provide it. Some respondents highlighted frustrations with patients who use the service for convivence.

“*Essential service for many patients but abused by others particularly since the Department of Health funded scheme*” (Participant 14)

“...*There is a risk of patients abusing the system, however, expecting prescriptions to be delivered out at a whim when drivers are already stretched*.” (Participant 37)

One respondent questioned the indirect impacts of delivery services perpetuating isolation.

“*Are we really helping people? Surely getting people out of their house and into society/the community this would help work against isolation*.” (Participant 1)

#### 3.2.2 Impact on pharmaceutical care

79% of respondents reported that delivery services increased adherence. All those that disagreed highlighted that lack of patient interaction which in turn reduced the ability of the pharmacist to provide comprehensive pharmaceutical care.

“*While it certainly makes it easier for patients to receive their medicines, I'm not sure if it affects compliance/adherence. The assumption is that patients who receive their deliveries take their medicines as prescribed, but it is difficult to say as we have no information regarding the monitoring of their condition*.” (Participant 34)

“*Compliance/Adherence may be affected both positively, due to easy access to medication, but also negatively due to less communication between patient and pharmacy*” (Participant 8)

“*It allows the pharmacy to ensure the patients receive their medication on specific days. This can help with patient compliance and adherence*.” (Participant 23)

“*...depends on the patient, some are not compliant regardless, but others are very appreciative of it.*” (Participant 12)

Overall, there was decreased face-to-face interaction, and this was felt to have a negative impact on care. Some respondents felt that the time taken to manage deliveries also reduced the time to deal with clinical problems which might present ad-hoc, negatively impacting patient care for many service users.

“*It's an extra level of administration which eats into normal day to day service but is helpful to many*.” (Participant 26)

“*They have a positive impact on the care for the patient receiving the delivery however the management and record keeping is very time consuming, and this reduces the time pharmacists and staff have for direct face to face care*.” (Participant 3)

“*[We] never have a chance to see or speak to these patients.*” (Participant 27)

Some participants identified that medicines delivery must contextualised within broader pharmaceutical care and that in some cases, particularly when the medicine is new, further counselling is required.

“*Yes [medicine delivery services do improve compliance], but only when part of a full medicines management service.*” (Participant 17)

“*Yes [medicine delivery services do improve compliance for] repeat medicines [but for] new medication no, further counselling is required.*” (Participant 22)

#### 3.2.3 Commercial impact

Only 30% of respondents agreed that delivery services demonstrate good value for the business. Although delivery services may assist vulnerable patients receiving their medicines, it also has a business impact on attracting and retaining customers; this was highlighted often by the participants.

“*Without remuneration none. Like many other aspects of community pharmacy, this is not driven by professionalism but by competition and commercialism*.” (Participant 17)

“*Few benefits to the pharmacy. Only offered by most to maintain competitiveness. Allows pharmacy to manage workload better. Peace of mind for pharmacist knowing that vulnerable patients receive medicines in timely manner*.” (Participant 31)

“*I feel that the current expectation of patients exceeds the cost effectiveness and time schedule available to pharmacy. A small charge for delivery would cut down on patients receiving them needlessly and provide more availability to the patients that truly require the service, and ensure counselling is fully conducted in branch.*” (Participant 29)

Some respondents suggested alternative funding modes for this service and were careful to consider the impact of unilateral funding mechanisms which may enable large multinationals to outcompete smaller independent pharmacies.

“*Unfunded Community Pharmacy Delivery Services have evolved from commercial competition... Funded delivery is essential, but it should be part of an HSCB Medicine Management Program, designed by pharmacists & HSCB, and regulated for professional assurance. As well as being patient centred, such a system would provide a level playing field between multiples & independents, since the service is based on a defined patient need, and not on commercial competition*.” (Participant 17)

“*Pharmacy delivery services, like MDSs, is a competitive tool allowing pharmacies initially to poach patients. Then it gets too expensive offered [Free of Charge] FOC so payment is sought. Now we are seeking payment and turning it into an essential service. It will stop the clinical development of pharmacy and community pharmacy will remain a supply service with little if any professional clinical input*.” (Participant 28)

#### 3.2.4 COVID on delivery services

COVID has seen a significant impact on the service.

“*The cost of the delivery service has spiralled since lockdown. It has become very difficult to refuse a deliver when someone is isolated or now afraid to come to the pharmacy. The cost of the service is not covered but (sic) any renumeration we receive*...” (Participant 3)

The additional funding provided to facilitate delivery services during the pandemic prompted one pharmacist to highlight problems with the current thinking around pharmacist renumeration.

“*...went from 10 charged deliveries per day to >150 free deliveries per day*.” (Participant 14)

“*During COVID pandemic Community pharmacies have received funding for delivery services, which has been welcomed by the profession, particularly smaller independents, who had been operating unfunded services out of necessity to patients. We are still only remunerated for dispensing prescriptions, we should be remunerated for medicine management, which is what we do! deliveries are part of this process*.” (Participant 17)

#### 3.2.5 Ethical, legal and safety risks

The lack of direct control of the pharmacist on the fate of the medicine once it leaves the pharmacy presents several ethical, legal and safety risks. Many respondents highlighted these risks with personal recollections.

“*Delivery to wrong address. Delivery failure of a critical medicine. Storage issues in warm environments. Pharmacy vans are target of criminals*.” (Participant 3)

“*All the usual of wrong person, address, or updated therapy/condition not communicated to pharmacy*.” (Participant 11)

“*Patient expectations that medicines can be popped through the letterbox when they are out.*” (Participant 6)

“*Turning community pharmacy into a take-away service like a [fast-food] restaurant*.” (Participant 28)

One respondent suggested that governance mechanisms can reduce these risks, but evidently these frameworks cannot eliminate them.

“*As long as [Standard Operating Procedures] SOP's are comprehensive and adhered to, risks can be managed appropriately. We have had very few problems*...” (Participant 37)

However, some of these risks are beyond the control of the pharmacy and their team.

“*Road traffic accidents to driver, delivery to incorrect patient, loss of direct contact with pharmacist, false claims that medicines not received*.” (Participant 33)

## 4 Discussion

### 4.1 Access to pharmaceutical care

Delivery services remove barriers and enable patients to receive their medicines in a consistent, timely fashion. This view is also seen in the literature with many authors describing how pharmacy delivery services increase patient access to medicines, particularly vital for the elderly and those in rural areas where a pharmacy may not be within walkable distance.^4^^,^^9^^,^^10^

A recent International Pharmaceutical Federation (FIP) report outlines a global perspective where access to pharmacists and pharmacies is highest in high economy countries and lowest in developing countries with rural areas.^26^ Globally, there is an average of 2.75 pharmacies per 10,000 people, with over 60% of pharmacies offering a delivery service.^26^ A Pharmacy Needs Assessment (PNA) published by Northern Irelands Health and Social Care Board (HSCB) identified that many pharmacies in Northern Ireland are ‘accessible’ (defined by 15 min drive in rural areas and < 1mile in cities; approximately equivalent to a 15-min walk) and they highlighted that the greatest need for pharmaceutical services is in predominately in cities.^27^ This disparity probably arises as our respondents perhaps do not see equality between 15-min walking and driving (i.e., the definition of accessible in the PNA above). The top 5 areas by greatest need: Crumlin (Co. Antrim), Magilligan (Co. Derry/Londonderry), Shankill (Co. Antrim), Parklake (Co. Armagh) and Derryaghy (Co. Antrim).^27^ Magilligan stands out with its high elderly population, a group identified as one with a very significant need for pharmaceutical services.^27^ Convenience was also a significant factor highlighted by the participants, which is perhaps easy to dismiss. However, evidence is emerging that can convenience brought about by home delivery services can translate to improved patient compliance.^9^^,^^28^

Quantifying the need for pharmaceutical services is an important step in the allocation of public funds. Although no audit can completely capture all aspects of care, the value of these services should be recognised where possible to ensure funding is provided where there is a need for it. This may be more challenging to define for interventions which have impacts on broader healthcare systems such those which seek to improve adherence (e.g., compliance aids and medication reviews). This places additional responsibility on health systems to provide the tools to enable data capture and on pharmacists to capture these activities, be they commissioned or non-commissioned. Further, this analysis (Table 2) suggests that the definition of access and the clinical context should be critically evaluated in each case to ensure patient needs are met. Equivalent access will not address inequivalent needs.

### 4.2 Quality of pharmaceutical care

Beyond access to medicines, some participants highlighted that delivery services may reduce the quality of care. Although, it could be argued that it is better for patients to receive their medicines than none, there are cases where comprehensive pharmaceutical care is essential. Our participants identified certain clinical situations (new medicines) and patient groups (the elderly) as those who require this additional care.

Time allotted to dispensing tasks reduces the time available for face-to-face care or for more complex clinical activities, this was experienced by several of our participants. This matter is compounded by increased risk of dispensing through home delivery services. While internet pharmacies dispense significantly larger volumes than the average community pharmacy, they perform very little additional services in comparison. When scaled for dispensing volume this gap becomes even more dramatic. Additional services such as the New Medicines Service or Medicines Use Review are targeted towards patients who are receiving complex care for conditions such as diabetes and asthma. These two services help patients understand how to take their medicines and why they have been prescribed them. The New Medicines Service helps patients who have been started on a new medicine and the Medicine Use Review scheme assists patients who have been prescribed drugs for a longer period of time. This, in addition to the views of pharmacists from this study which suggested that home delivery services are not cost-effective, which may be indicative of different operating structures. In contrast to the views of the pharmacists in this study, the proliferation of internet pharmacies suggests that a business model centred around home delivery is viable. However, when one attempts to squeeze that dispensing volume into a traditional community pharmacy format it may not fit. The participants identified that it is difficult to provide standard care and as such this may represent an opportunity to reallocate pharmacist time. As pharmacists and their teams take on new responsibilities – some of which are now commissioned – such as New Medicines Service and Community Pharmacy Consultation Service, this may provide an opportunity to delegate dispensing tasks away from the pharmacy (once a clinical check has been performed) to enable the development of these new services.

The Pharmacy Needs Assessment exclusively focused on dispensing data^27^ – perhaps a minimum standard within the context of modern pharmaceutical care – audits of this sort cannot account for all aspects of care, particularly where it is non-commissioned. This approach has been critically evaluated in other areas of healthcare.^29^^,^^30^ Referring to the definition of pharmaceutical care provided by Hepler and Strand i.e., “*the responsible provision of drug therapy for the purpose of achieving definite outcomes that improve a patients' quality of life*.”^31^ Home delivery services can *enable the responsible provision of drug therapy*, but they may struggle to export other aspects of patient care which monitor the *definite outcomes that improve a patients quality of life*. Digital health can improve access to these additional services but, as seen with other healthcare professionals, an online consultation cannot replace therapeutic presence^29^^,^^30^ and within the pharmacy context, some services are more difficult to export e.g., counselling for more complex formulations such as inhalers, nebulisers and anti-diabetic medicines.

Globally, a report by the FIP highlighted that the majority (51%) of pharmacies across the globe are remunerated through product based models,^26^ this funding model will disincentivise pharmacists to provide access to comprehensive pharmaceutical care. As identified by Hepler and Strand in 1990, the pharmaceutical care system in pharmacy is yet to facilitate patient access to comprehensive pharmaceutical care.^31^

### 4.3 Balancing commercial pressure with professional obligation

This study has highlighted – not for the first time – a tension between the professional obligations and commercial pressures that exist within community pharmacy. Some participants reflected that home delivery was not a professional service and only emerged to attract patients. This approach is perhaps underpinned by the current reimbursement model which directs funds towards dispensing output as opposed to the quality of patient care. This theme has been identified previously, as pharmacists are disincentivized to provide resource expensive additional services particularly when they have an ethical and professional responsibility for their pharmacy team to prevent stress and overwork while keeping the business competitive and sustainable.^1^ Some participants described the use of a fixed charge for delivery services. Presumably they assume that patients who value this service will pay the charge to have medicines delivered. However, for the patients who do not have the means to pay, refusal to pay does not reflect the value they place on the service; is access to pharmaceutical care something that should not be for sale?^32^

To find this balance, many participants expressed a need for further funding and participants were careful to highlight that a different model would need to be in place for larger pharmacy groups (including internet pharmacies) and smaller independent pharmacies as the current funding paradigm does not offset the cost. This is particularly complex with the arrival of online pharmacies; a funding scheme will need to be carefully tailored or perhaps a new model may be appropriate as automation becomes more prominent – this area is in critical need of further study.

### 4.4 Risks and risk management

The lack of direct oversight by the pharmacist at the point of medication exchange increases risks which has prompted the development of standards and recommendations by the professional regulatory bodies internationally.15., 16., 17., 18, 19. However, it appears that this guidance may be at odds with what is practically feasible in a community pharmacy environment, particularly with the increases in workload that come with offering a delivery service; the biggest workload relates to the management of this risk. Further, the General Pharmaceutical Council (GPhC) identify medicines that require additional safeguards,^33^ but they do not detail what these safeguards would look like. Questions arise, are there medicines that should not be dispensed online and require direct pharmacist oversight? What would remote pharmaceutical care in that context look like? Should the pharmacist alone make the delivery and assess the patient understanding at that point?

Participants also highlighted that convenience may trivialise the importance of pharmaceutical care, particularly as some patients request that their medicines are placed through the post-box; one participant drew comparisons to a fast-food delivery service. One of the responses suggested that medicines supply is almost automatic, and this caused problems when there were changes made to the therapeutic regimen, which was not communicated to the pharmacy. These risks may be compounded by emerging digital tools whereby the interaction with healthcare professionals can be conducted entirely online, and in some cases, by a physician in a different country.

### 4.5 Limitations and areas for further research

We found that we obtained a reasonable distribution of responses similar to the distribution of pharmacies in NI but we note a lack of responses from delivery drivers and no responses from pharmacy teams in Fermanagh, the most rural county. Problems highlighted in this study with regards to access might be compounded in this region. We also note that we do not have data from internet pharmacies, where significantly large proportions of pharmacy services were delivered online, which may have a different dispensing model. As such, we would expect that their views may differ from the traditional brick-and-mortar pharmacy found in this study, particularly with respect to the issues highlighted such as managing risk and their throughs on quality pharmaceutical care. This study does not make generalisations about pharmacy delivery services as our aim was to simply probe this under explored area of pharmacy practice to highlight areas for further study (which is ongoing).

## 5 Conclusion

Changes to dispensing behaviour in community pharmacies has historically been a major driver of change within the profession, a modern driver in this context is rapid growth of dispensing volume within online pharmacies. Despite this, there remains a critical need for research in community pharmacy to evaluate current and emerging modes of practice and identify areas for improvement. To our knowledge, this is the first study of its kind to approach the topic of remote pharmaceutical care delivered by community pharmacies.

We find that pharmacy teams saw delivery services as a means to improve access to pharmaceutical care, increasing adherence particularly for the elderly. But this activity limits the time available for comprehensive pharmaceutical care. Clearly, remote pharmaceutical care can be provided in a safe and effective manner and is a valuable service for our communities. This service is most suitable for patients who are compliant, well managed and without complications. For this patient, internet pharmacies reimbursed by the current dispensing model may be adequate. However, there is a critical need for researchers to explore if another reimbursement model is perhaps more appropriate for traditional brick-and-mortar pharmacies as they transition into a public health role and as prescribing becomes part of the toolkit of every pharmacist, particularly in the UK.

As identified by participant 34, future research should gauge patient views of this service to address the variety of questions that have been identified in this work: is remote pharmaceutical care a matter of convenience? Does remote pharmaceutical care help patients adhere to their medicines? How do digital health innovations impact care across patient demographics? What does comprehensive pharmaceutical care mean for patients?

## CRediT authorship statement

OK was responsible for conceptualisation and writing the manuscript for publication, AC and DL managed the ethics application and FK preformed a preliminary literature analysis and AC and DL administered the survey. OK AC and DL received funding and were responsible for supervision of the project. All authors agreed to the contents of the final manuscript prior to submission.

## Declaration of Competing Interest

There are no conflicts to declare.
